# Determination of Glyoxalase-1 levels and Identification of Genetic Variants in *GLO1* Gene in Patients of Diabetic Nephropathy

**DOI:** 10.12669/pjms.40.4.8258

**Published:** 2024

**Authors:** Syed Zubair Hussain Shah, Amir Rashid, Asifa Majeed

**Affiliations:** 1 Syed Zubair Hussain Shah, FCPS. Associate Professor of Biochemistry, Department of Biochemistry & Molecular Biology, Army Medical College, National University of Medical Sciences, Rawalpindi, Pakistan; 2 Amir Rashid, PhD. Professor, HoD Biochemistry, Department of Biochemistry & Molecular Biology, Army Medical College, National University of Medical Sciences, Rawalpindi, Pakistan; 3 Asifa Majeed, PhD, Post Doc. Associate Professor of Biochemistry, Department of Biochemistry & Molecular Biology, Army Medical College, National University of Medical Sciences, Rawalpindi, Pakistan

**Keywords:** Methylglyoxal, Glyoxalase-1 (GLO1), Diabetic Nephropathy, Advanced Glycation End products (AGEs), SNP rs4746

## Abstract

**Objective:**

To determine the association of diabetic nephropathy with glyoxalase-1 enzyme levels and a genetic missense variation (SNP rs4746) in its gene (GLO-1).

**Methods:**

This cross-sectional comparative study was conducted at the Department of Biochemistry and Molecular Biology, Army Medical College, Rawalpindi from November 2020 to December 2022. One hundred patients and one hundred and thirteen healthy controls were enrolled using the nonprobability convenience sampling method. Medical history and 10ml blood were obtained from each individual after written informed consent. Blood samples were subjected to biochemical tests and DNA extraction which was later used for single nucleotide polymorphism (SNP) analysis (C332C variant of rs4741 *GLO*-1 gene) using Tetra primer ARMS PCR and gel electrophoresis. Glyoxalase-1 enzyme activity in serum was measured using ELISA.

**Results:**

There was a significant difference in serum glyoxalase-1 levels in the two groups *(p-*value< 0.001). The patient group had lower levels (16.24 ± 22.51mg/dl) of glyoxalase-1 as compared to the control group (48.70 ± 42.54mg/dl). In genotypic analysis, 98 out of 100 control individuals had AA genotype-while only one had CC and another AC genotype. In the patient group, 94 out of 100 patients showed AA genotype, three AC, and three CC genotypes. As the statistical significance (*p*-value) was 0.37, there was no significant association found between AC or CC genotype and diabetic nephropathy.

**Conclusion:**

Glyoxalase-1 levels are linked to the development of diabetic nephropathy in our patients while a known missense variant rs4746 in the GLO-1 gene is not associated with increased risk.

## INTRODUCTION

Diabetes mellitus (DM) is one of the most prevalent diseases globally and the magnitude of the problem is expected to rise globally from 10.5% (236.6M) in 2021 to 12.2% (783.2M) by 2045 with almost half of the patients going undiagnosed. Cost of healthcare will increase from 966Bn USD in 2021 to 1054Bn USD in 2045.^1^ Diabetic nephropathy (DN) is a major known complication of diabetes and a leading cause of morbidity & mortality in type-1 and type-2 diabetics worldwide^2^ including Pakistan.^3^,^4^ It ultimately leads to end-stage renal disease (ESRD) and there is no cure other than hemodialysis or renal transplant. It starts with increased creatinine levels and finally decreased glomerular filtration rate (GFR).^5^ There have been two clinically distinct patterns of diabetic nephropathy observed in patients with diabetes mellitus.^6^

In one type-patients show milder disease, present with micro-albuminuria and a mild increase in creatinine levels, respond well to the treatment, and remain problem free for years.^6^ While the second group patients show up with significant renal impairment early in the course of disease and go quickly into uncompensated end-stage renal disease. The patients in the second group need continuous dialysis or a renal transplant.^6^ Inherited factors have been implicated in the development of diabetes and diabetic nephropathy in addition to lifestyle and eating habits^7^. In this study, our focus was to look for any possible genetic variations having a positive correlation with early diabetic nephropathy.

The percentage of patients with diabetes developing nephropathy and the percentage of those developing a severe form of it, greatly vary among different ethnicities, pointing towards a genetic factor as a predisposing factor.^8^ The heritability of diabetic kidney disease is 35% in type-1 diabetics and has been linked even more with the genes related to type-2 diabetes.^9^ Through using Genome-Wide Association Studies (GWAS) and Next Generation Sequencing (NGS), a few single nucleotide polymorphisms (SNPs) have been found and their related genes have been investigated, however, no genetic cause of the disease has been established yet.^8^

In 2020, Spanish and German research laboratories have reported a few genes related to glutathione metabolism with development of nephropathy but not diabetic nephropathy.^10^ Clinically evident nephropathy occurs in 20-50% diabetics within 5-10 years of start of disease.^8^,^11^ Almost 25% out of 50% developing diabetic nephropathy (marked by microalbuminuria & (urinary albumin to creatinine ratio) uACR 30-300mg/g/day, eGFR > 90 ml/min/1.73 m2) exhibit frank proteinuria and advanced renal failure (uACR >300mg/g, eGFR < 60 ml/min/1.73 m2 to < 15 ml/min/1.73 m2) despite use of angiotensin receptor blockers (ARBs) or angiotensin converting enzyme (ACE) inhibitors.^3^

The underlying mechanism of diabetic nephropathy (DN) is still not fully under-stood, however advanced glycation end products (AGEs) are thought to play a major part.^12^ Methylglyoxal (MG) is a by-product of glycolysis and an inducer of AGEs formation.^13^ Glyoxalase-1 (*GLO1*) is a cytosolic enzyme present in most of the cells which continuously degrades MG to reduce the formation of AGEs.^13^ SNP rs4746 (C332C) polymorphic form of *GLO1* gene may lead to production of a slower isoform of glyoxalase-1 enzyme probably leading to early and severe form of DN.^14^,^15^ The SNP rs4746 (previously denoted as rs386572987) is one of the most important missense variations reported in the gene with clinical consequences including Type-2 diabetes mellitus (T2DM) though not diabetic kidney disease.^16^ There is a change from glutamate to alanine at position 111 (Glu111Ala) as a result of A mutated to C or T (T changed to G or A in the forward strand) with mean allelic frequency (MAF) C=0.36.^16^

The subset of diabetic nephropathy leading to ESRD quickly, without relevant clinical proteinuria has not yet been studied for underlying genetic basis. If an association is found between a specific polymorphism in this gene and a severe and early onset of diabetic nephropathy, it will help further research on this aspect.

## METHODS

This cross-sectional comparative study was conducted at the Center for Research in Experimental and Applied Medicine (CREAM), Department of Biochemistry & Molecular Biology, Army Medical College, Rawalpindi in collaboration with the Department of Nephrology Pak Emirates Military Hospital (PEMH), and Armed Forces Institute of Transfusion (AFIT) Rawalpindi. This was a case-control study conducted from November 2020 to December 2022. A total number of 213 individuals were enrolled using non-probability convenience sampling, out of which one hundred were healthy controls and one hundred and thirteen were suffering from advanced renal disease developed within ten years of diagnosis of diabetes. The disease group included patients of both Type-1 and 2 diabetes, aged 18 to 80 years with glycosylated hemoglobin (HbA1c) > 6.5% or 48mmol/mol.

In addition, the patients were selected who had less than ten years of diagnosis of diabetes with eGFR < 45ml/min/1.73 m_2_^17^ or who were put on dialysis within ten years of diagnosis of diabetes.^17^ In the control group, 18-80-year-old healthy adults without any disease especially related to glucose homeostasis and kidneys, were enrolled having HbA1c 6.5% (or 48mmol/mol) or less and urea and creatinine within normal limits. Unconscious patients and those having comorbidities like hypertension, recent illness, or who were taking any other drug, were excluded. Approximately 10mL of blood samples were taken (using two tubes BD vacutainers, one for serum the other with EDT for blood) and preserved for chemical and genetic analysis. These samples were used for HbA1c estimation through ELISA, biochemical tests and gene polymorphism. DNA extraction was carried out by both, standard phenol-chloroform method^18^ and DNA extraction kit method (Favorgen Taiwan) and quality was checked on 1% agarose gel. Primers were prepared using primer three plus software (available at https://www.bioinformatics.nl/cgi-bin/primer3plus/primer3plus.cgi) and tetra primer ARMS PCR was conducted after acquiring primers from Macrogen company.

**Table:** 

Primer	Sequence	Melting Temp ^o^C
Forward inner	GGGACTGAAGATGAGGG (G Allele)	59
Reverse inner	TGTGGTAACTCTGGGGCT (A Allele)	56
Forward outer	AATCATACTTTGGCCATTTGTC	57
Reverse outer	TGATCATACTTAATTAGGACATTAATGTT	57

### Ethical Approval

The ethical review committee (ERC) of the university/ institution approved the study vide letter number ERC/ID/96 dated 12 November 2020 and written informed consent from the patients was taken before the start of the study.

PCR results were analyzed using ethidium bromide stained gel electrophoresis with 2% agarose gel. Urea & creatinine were measured using fully automatic chemistry analyzer based on the principle of a spectrophotometer (Roche Cobas E411). While HbA1c and glyoxalase-1 levels were measured using ELISA. eGFR was calculated using MDRD equation which is 186 x (Creatinine/88.4)^-1.154^ x (Age)^-0.203^ x (0.742 if female) x (1.210 if black).^19^ The primers designed for tetra primer ARMS PCR using primer three plus software are given in the table below. The albumin was estimated by a photometric method using an Albumin estimation kit by Sigma Aldrich.

The protocol and temperature settings were optimized (it worked best at temp 58^o^C) in a few trials and then samples were checked using PCR and gel electrophoresis in batches of 7 or 14 samples. A 100bp ladder was used to identify our fragments of interest. Biochemical data was analyzed by SPSS version 22. Allelic frequency, genotypic frequency, and Hardy Weinberg equilibrium were calculated using the biological software SNPstat. The Chi-Square test (X^2^) was used to estimate the significance of the differences among the groups. A *p-value* of ≤ 0.05 was considered significant. The comparison of the means of baseline investigations to ascertain cases versus controls is mentioned in Table-I.

**Table-I:** Mean values of biochemical parameters in two groups.

Parameter	Group	N	Mean ± SD
HbA1c (%)	Control	100	5.35 ± 0.56
	Patients	113	8.04 ± 1.17
Urea (mmol/l)	Control	100	4.28 ± 1.00
	Patients	113	23.85 ± 15.08
Creatinine (mmol/l)	Control	100	88.23 ± 17.32
	Patients	113	487.63 ± 252.54
eGFR (ml/min)	Control	100	102.16 ± 23.60
	Patients	113	14.30± 18.50
Albumin (g/dl)	Control	95	48.24 ± 5.27
	Patients	76	38.84 ± 7.57

## RESULTS

**Fig.1:**
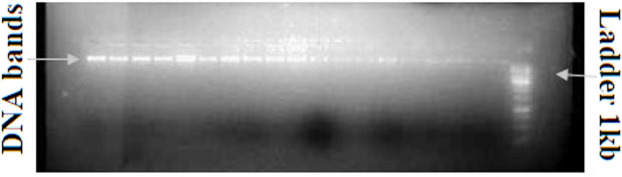
Image of DNA Electrophoresis on 1% Agarose Gel.

**Fig.2:**
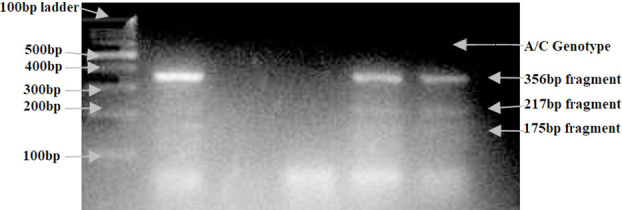
In heterozygous patients, A/C alleles on Gel after tetra ARMS PCR.

There was a significant difference in serum glyoxalase-1 levels in the two groups *(p-*value< 0.001). The patient group had lower levels (16.24 ± 22.51mg/dl) of this protective enzyme (glyoxalase-1) suggesting a possible association with diabetic nephropathy, as compared to the control group (48.70 ± 42.54mg/dl) shown in Table-II. In genotypic analysis (tetra primer ARMS PCR product gel electrophoresis), 98 out of 100 control individuals had AA genotype-while only one had CC and another AC genotype. In the patient group, 94 out of 100 patients showed AA genotype, three AC, and three CC genotypes. As the *p-value* was 0.37, there was no significant association found between AC or CC genotype and diabetic nephropathy. The product size obtained for G allele was 175bp, for A allele 217bp, and for the whole product of two outer primers was 356bp. The DNA bands obtained on gel electrophoresis are depicted in Fig.1 below while Fig.2 shows bands of tetra primer ARMS gel electrophoresis showing heterozygous pattern in patients. Genotypic frequency is shown in Table-III while mean allelic frequency in our study groups and the one noted globally for risk allele (C) in other diseases is shown in Table-IV.

**Table-II:** Comparison of Glyoxalase-1 serum levels in two groups.

Parameter	Group	N	Mean ± SD (mg/dl)	p-value
Glyoxalase-1	Control	81	48.70 ± 42.54	< 0.001
	Patients of Diabetic Nephropathy	85	16.24 ± 22.51	

**Table-III:** Association of rs4746 genotype-with diabetic nephropathy.

Gene/ snp	Genotype	Patients of DN [n(%)]	Healthy Controls [n(%)]	p- value
GLO-1/ rs4746	AA	94 (94%)	98 (98%)	0.37
	AC	3 (3%)	1 (1%)	
	CC	3(3%)	1 (1%)	

**Table-IV:** Mean allelic frequency in two groups.

Gene/ snp	Genotype-	Patients of DN [n(%)]	Healthy Controls [n(%)]	p- value
GLO-1/ rs4746	AA	94 (94%)	98 (98%)	0.37
	AC	3 (3%)	1 (1%)	
	CC	3(3%)	1 (1%)	

## DISCUSSION

This study found a positive correlation between serum glyoxalase-1 enzyme levels and the development of diabetic nephropathy in the Pakistani population. Several other studies have suggested a role of this enzyme in risk of different diseases including diabetes mellitus.^20^,^21^ Dysfunctional glyoxalase-1 has also been implicated in development of nephropathy through decreased degradation of methylglyoxal and subsequently formation of more AGEs.^22^ Magniferin, an inducer of glyoxalase-1, is reported to lower the formation of AGEs and prevent diabetic nephropathy.^23^ So variations in serum glyoxalase-1 enzyme levels were suspected to be associated with the phenomenon of early and severe diabetic nephropathy in some patients. Affirmatory results point towards the role of this enzyme in the development of diabetic nephropathy. This is reported first time in the Pakistani population and can be a lead forward toward personalized treatment in the future.

After obtaining results indicating a significant association between glyoxalase-1 levels and diabetic nephropathy, we explored a specific genetic variation possibly playing a role in this disease. *GLO-1* gene (which encodes glyoxalase-1 enzyme) has not been explored well for an association of its variations with the development of diabetic nephropathy. In the literature, some other genes have been explored for association with DN e.g. (1) Toll-like receptor *(TLR*) gene mutations have been linked with the development of kidney disease,^24^ (2) Angiotensin-converting enzyme (*ACE*) insertion-deletion polymorphism has also been linked with the development of diabetic nephropathy^25^ (3) Another study looked for endothelial nitric oxide synthase gene (*G894T, 4b/a, and T786C*) polymorphism and development of diabetic nephropathy.^26^ Genes, for example, that for catalase, related to oxidative stress are associated with diabetes and nephropathy independently.^27^

A recent study has reported the involvement of pro-inflammatory cytokines interleukin-16 and interleukin-18 gene expression with the development of diabetic nephropathy but it does not comment on the severity or early onset of disease.^28^ There is no significant association found between these polymorphisms and the severity of early diabetic nephropathy except for a few genes in certain populations. Based on the discussion above it is evident that there was a need to explore polymorphism in more genes related to renal function like *GLO-1, CBR-1*, and *ACE*, and look for any association with the development of early severe diabetic nephropathy.

We shortlisted the most common SNP in the *GLO-1* gene i.e. rs4746 which is reported to have clinical outcomes as well e.g. autism.^29^ A change of A to C in rs4746 (re-named from rs2736654) changes alanine to glutamic acid at amino acid position 111. In this study, a relationship couldn’t be established between any specific polymorphic form of rs4746 in the *GLO-1* gene and early development of nephropathy in diabetes in the Pakistani population. However, it is observed that if *GLO-1* is induced, it abolishes the changes that occur in diabetic nephropathy and improves the outcome^29^. This points out a need to look for other variations in this gene which leads to relatively lower quantity or function of enzyme in early developers of diabetic complications.

### Limitations of the study

Due to lack of funds we could not enroll an even bigger sample size and could not include more suspected gene variation.

## CONCLUSION

The glyoxalase-1 enzyme levels are deranged in patients afflicted with severe and early diabetic nephropathy. However, polymorphism at rs4746 is not found to be associated with this change in serum glyoxalase-1 levels. So other polymorphic sites/ forms of *GLO-1* gene may be explored for association/ causation.

### Future recommendations

Replication of the study with large cohorts is suggested to further confirm the results.

### Authors Contribution:

**SZHS**: Designed and performed the study including proposal writing, data collection, performance of lab tests and genetic analysis.

**AR**: Designed & contributed in manuscript writing. He is responsible for integrity and accuracy of the study.

**AM**: She did review of manuscript, helped in literature search and provided help and technical supervision/ guidance in genetic analysis.
